# Acute Myeloid Leukemia-Related Proteins Modified by Ubiquitin and Ubiquitin-like Proteins

**DOI:** 10.3390/ijms23010514

**Published:** 2022-01-03

**Authors:** Sang-Soo Park, Kwang-Hyun Baek

**Affiliations:** Department of Biomedical Science, CHA University, Seongnam-si 13488, Gyeonggi-do, Korea; sangsoo3943@naver.com

**Keywords:** deubiquitination, ISGylation, NEDDylation, SUMOylation, ubiquitination

## Abstract

Acute myeloid leukemia (AML), the most common form of an acute leukemia, is a malignant disorder of stem cell precursors of the myeloid lineage. Ubiquitination is one of the post-translational modifications (PTMs), and the ubiquitin-like proteins (Ubls; SUMO, NEDD8, and ISG15) play a critical role in various cellular processes, including autophagy, cell-cycle control, DNA repair, signal transduction, and transcription. Also, the importance of Ubls in AML is increasing, with the growing research defining the effect of Ubls in AML. Numerous studies have actively reported that AML-related mutated proteins are linked to Ub and Ubls. The current review discusses the roles of proteins associated with protein ubiquitination, modifications by Ubls in AML, and substrates that can be applied for therapeutic targets in AML.

## 1. Introduction

Acute myeloid leukemia (AML) is a malignant disorder of hematopoietic stem cells, and the most common form of an acute leukemia. It is characterized by clonal expansion of abnormally differentiated blasts of the myeloid lineage in adolescents and the young adult population, having an increasing incidence with advancing age [1,2,3]. Despite these common characteristics, the disease is highly heterogeneous, and patients exhibit significant differences in genetic abnormalities, paracrine and autocrine growth regulation, and transcriptional regulation, as well as proteomic and cellular metabolomic profiles [4]. In the United States, 19,940 new cases were diagnosed in 2020, with about 11,180 fatalities reported [5]. AML is a relatively rare cancer, constituting 30% of all leukemia cases and about 1.1% of all cancers in the United States [6]. Although the median age of diagnosis is 68 years, AML can be detected at any age [6].

The numerous gene mutations found in AML have been identified using the next generation sequencing (NGS) technology (Table 1). Molecular detection of gene mutations has an increasingly important role in classification, risk stratification, and management of AML. The three most common gene mutations found in AML are FMS-like tyrosine kinase 3 (FLT3), nucleophosmin 1 (NPM1), and DNA methyltransferase 3α (DNMT3α). FLT3 is a receptor tyrosine kinase that plays an important role in processes such as differentiation, proliferation, and survival in hematopoietic cells [7,8]. The *FLT3* mutations occur in approximately 30% of all AML cases and are a poor prognostic factor for the patients [7]. There are two major types of FLT3 mutations. The internal tandem duplication (FLT3-ITD) mutations represent the most common type of *FLT3* mutation, occurring in approximately 25% cases, whereas the point or deletion mutations in the tyrosine kinase domain (FLT3-TKD) occurs in approximately 7~10% cases [9]. The *NPM1* mutation occurs in up to 30% AML patients [10], and is related to a higher probability of complete remission, improved overall survival, and a lower cumulative incidence of relapse [11]. DNMT3α is a DNA methyltransferase involved in the epigenetic regulation of the genome through methylation. The *DNMT3*α mutations occur in approximately 20% AML patients and relate with a poor prognosis [12,13]. Other gene mutations of AML include Tet methylcytosine dioxygenase 2 (TET2), RAS, and CCAAT enhancer binding protein α (C/EBPα). TET2 regulates cell fate decisions during development and in embryonic stem cells, by maintaining the pluripotency or by regulating differentiation through catalyzing the demethylation of 5-methylcytosine on DNA [14]. *TET2* mutations occur in approximately 10~20% AML patients and impart a variable prognosis, depending on the presence of additional pathogenic events [15]. The *RAS* family gene mutations (*NRAS* and *KRAS)* are found in 10~15% AML cases [16]. These gene mutations result in aberrant proliferative signaling through the RAS/RAF/MEK signaling pathway [16]. C/EBPα is a transcription factor that plays an important role in the lineage-specific myeloid differentiation, and *C/EBP*α mutations occur in ~10% AML cases [17].

Ubiquitination is one of the post-translational modifications which is critical for a number of cellular processes such as protein degradation, cell cycle progression, transcriptional regulation, DNA repair, and signal transduction [18]. Ubiquitin (Ub) is covalently bound to lysine residues on target substrates by an enzymatic cascade accomplished by the sequential activation of the activating (E1), conjugating (E2), and ligating (E3) enzymes [19]. Ub is first activated by the E1 activating enzyme and is transferred onto an E2 conjugating enzyme [19]. Subsequently, E3 ligases interact simultaneously with the ubiquitin-loaded E2 and the target protein, and induce an isopeptide bond between the C-terminus of ubiquitin and lysine residue of the substrate [20].

**Figure 1:**
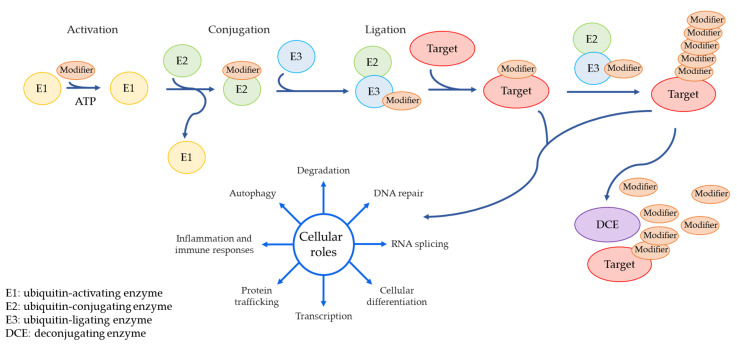
An enzymatic cascade of Ub and Ubls, and deconjugating enzymes. Ub and Ubls are conjugated to target substrates by an enzymatic cascade involving E1, E2, and E3. A modifier is first activated by an E1 activating enzyme and is transferred onto an E2 conjugating enzyme. Subsequently, E3 ligase interacts simultaneously with a modifier-loaded E2 and the target protein, and induces an isopeptide bond between the C-terminus of modifier and a lysine residue of the substrate. Once a modifier is conjugated to their targets, polypeptide chains are formed due to the presence of suitable motifs on modifiers. Deconjugating enzymes are responsible for the reverse process of the modification.

Ub and the ubiquitin-like proteins (Ubls) bind to proteins and modify the stability, conformation, and subcellular localization of the target [21]. About a dozen Ubls have been identified in humans, including the small ubiquitin-like modifier (SUMO), the neuronal precursor cell-expressed developmentally down-regulated protein 8 (NEDD8), and the interferon-stimulated gene 15 (ISG15) [22]. Ubls are also conjugated to target proteins by an enzymatic cascade involving a Ubl E1, a Ubl E2, and typically a Ubl E3 (Figure 1) [22]. At least 8 distinct Ubls (including ubiquitin) have been demonstrated to modify cellular proteins (Table 2), and along with several other Ubls suspected of possessing similar ability. Although some Ubls (such as SUMO) regulate a number of substrates similar to ubiquitin, most Ubls have a far more limited range of substrates. SUMO has three major protein isoforms: SUMO1, SUMO2, and SUMO3 [23]. SUMO2 and SUMO3 are almost identical in their amino acid sequences (95% homology), whereas they share about 50% identity with SUMO1 [24]. SUMO2/3 are able to undergo polySUMOylation with their substrates. However, SUMO1 can only form mono-SUMOylation [25]. In human, there are six autophagy-related protein 8 (ATG8) proteins that are expressed at considerable levels, which are subdivided into the LC3 (LC3A, LC3B, and LC3C) and GABARAP (GABARAP, GABARAPL1, and GABARAPL2) families [26]. Activation of ATG8 is catalyzed by ATG7 (ATG8 E1) [27], following which ATG8 is transferred to ATG3 (ATG8 E2) [27]. Finally, the ATG12-ATG5-ATG16 complex acts as an ATG8 E3 ligase [27]. FAT10ylation is catalyzed by an E1 (UBA6), an E2 (USE1), and an E3 (not found yet) enzyme [28]. FAT10 consists of two Ubl domains combined by a linker. The N-terminal domain of FAT10 is 29% identical to ubiquitin and the C-terminal domain displays a 36% identity to ubiquitin. Small ubiquitin-like archaeal modifier protein 1 (SAMP1) and small ubiquitin-like archaeal modifier protein 2 (SAMP2) were first identified in archaea (*Haloferax volcanii*) [29]. E1-like SAMP-activating enzyme of archaea (UbaA) appears to function in SAMPylation similar to E1 enzymes [30]. However, SAMPs E2 and E3 have not been identified in the majority of archaeal genomes. A homology research in yeast first identified the ubiquitin related modifier 1 (URM1) together with its E1 enzyme, the ubiquitin-like activating enzyme 4 (UBA4) [31]. However, the mechanism of the URM1 and UBA4 system remains unresolved.

Deconjugating enzymes (DCEs) include the deubiquitinating enzymes (DUBs) and Ubl-specific proteases (ULPs) [35]. DCEs are involved in the cleavage of peptide, amide, ester, or thioester bond at the C-terminus of Ub and Ubl, and also in reversing the modification of target proteins by a mono Ub or Ubl and polyubiquitin or poly-UBL chains on target proteins [36].

There are numerous ongoing researches on Ub and Ubls, and the results of the studies on Ub and Ubls associated with AML are also increasing. In the current review, we discuss the cellular functions of proteins associated with Ub and Ubls in AML.

## 2. Ubiquitination in AML

The conjugated ubiquitin forms a polyubiquitin chain with additional ubiquitins. The conjugation sites are seven lysine residues (K6, K11, K27, K29, K33, K48, and K63) or methionine 1 (M1) residue of ubiquitin [18]. It has been demonstrated that cellular functions of protein substrates vary depending on which residue of the polyubiquitin chain is involved. The K6-linked polyubiquitin chain is involved in mitophagy and DNA repair [37]. The K11-linked polyubiquitin chain controls the cell cycle, proteasomal degradation, mitophagy, trafficking, and endoplasmic reticulum-associated protein degradation [38]. The K27-linked polyubiquitin chain activates several kinases and regulates DNA repair [39]. The K29-linked polyubiquitin chain plays a role in a kinase modification and proteasomal/lysosomal degradation [40]. The K33-linked polyubiquitin chain induces modification of kinases, innate immunity, and autophagy [41]. The most well-known are the K48-linked polyubiquitin chain and the K63-linked polyubiquitin chain, which induce proteasomal degradation and play a role in protein kinase signaling and DNA damage response, respectively [42,43]. Finally, the M1-linked polyubiquitin chain activates immune signaling and NF-κB activation [44].

The ubiquitin E3s are the most diverse enzymes in the ubiquitin system (there are about 650 ubiquitin E3s in humans), contributing to the specificity of the ubiquitination system [45]. Based on the existence of characteristic domains and the mechanism of ubiquitin transfer to the target protein, the ubiquitin E3 ligases are classified into three main families: really interesting new gene (RING), homologous to E6AP C-terminus (HECT), and RING-between-RING (RBR) [45]. RING is characterized by the existence of a zinc-binding domain called RING [46]. The RING domains are responsible for binding the Ub-conjugated E2 and stimulating the ubiquitin transfer [46]. The RING domains bind to target proteins and E2 enzymes and directly transfer the ubiquitin from the Ub-conjugated E2 to the isopeptide bond of lysine residues in the substrate [46]. The HECT E3 characteristic is existence of a HECT domain that catalyzes the ubiquitin transfer to the target protein through a two-step reaction [47]. The HECT domain receives the ubiquitin from the Ub-conjugated E2, and subsequently transfers the ubiquitin to target proteins [47]. RBR E3 is characterized by the existence of a RING1-in-between-ring-RING2 motif (such as HECT E3s), and is involved in catalyzing the ubiquitin transfer through a two-step reaction [48]. The RING1 domain recruits Ub-conjugated E2, transfers the ubiquitin to the catalytic cysteine of the RING2, and finally transfers to the substrate [48]. There also exist multi-subunit E3s such as the Skp1-Cullin-F-box protein complex (SCF complex). The SCF complex is composed of three constant components: RING-box 1 (RBX1) which is a RING-finger protein that recruits the Ub-conjugated E2, cullin 1 (CUL1) which is a scaffolding protein, and the S-phase kinase-associated protein 1 (SKP1) which is an adaptor that bridges the SCF complex with various F-box proteins, bringing the target specificity of the SCF complex and its corresponding target proteins [49]. The SCF complex is classified into three main families based on their substrate recognition domains: the F-box and WD repeat domain containing (FBXW), F-box and leucine-rich repeat (FBXL), and F-box only with uncharacterized domains (FBXO) [49].

### 2.1. Ubiquitin-Conjugating Enzyme E2 E1 (UbE2E1 also Known as UbcH6)

UBE2E1 is a sub-family of E2E1 that belongs to the class III E2 enzymes [50]. According to the analysis of two independent data sets of patient samples, eight AML prognostic genes were identified, including *ACOT11*, *FAXDC2*, *FECH*, *HBD*, *KLF1*, *LEF1*, *SLC25A37*, and *UBE2E1* [51]. However, the expression of only one gene (*UBE2E1*) was associated with the overall survival of AML [51], and low *UBE2E1* expression was related to a better outcome [51]. *UBE2E1* expression was also related to chemotherapy, and patients with relatively higher *UBE2E1* expression were more likely to be unresponsive to chemotherapy [51].

### 2.2. Casitas B-Lineage Lymphoma (c-Cbl)

c-Cbl is a member of the CBL family, which is a single-subunit RING E3 [52]. FLT3 is one of the receptor tyrosine kinases and is restrictedly expressed in the hematopoietic compartment [53]. c-Cbl is a Ub E3 ligase, which ubiquitinates and downregulates FLT3-ITD in AML [54]. Sequencing analysis of Cbl in blasts obtained from AML patient bone marrows revealed one case having a *c-Cbl* point mutation (Cbl-R420Q) which is unable to function as a Ub E3 ligase of FLT3 [54]. Moreover, the *c-Cbl* mutation (R420Q) disturbs FLT3 signal mitigation. Conversely, downregulation of FLT3-ITD by c-Cbl interferes with the function of negative regulators of receptor tyrosine kinase (RTK) signaling in vivo [55].

### 2.3. Casitas B-Lineage Lymphoma Proto-Oncogene-b (Cbl-b)

Cbl-b is a member of a conserved E3 ubiquitin ligase Cbl family, and is involved in downregulating the signaling of activated RTKs [56]. Cbl-b ubiquitinates and downregulates SIVA1, a Ub E3 ligase of another tumor suppressor (ARF) that is a positive regulator of p53 [57], which in turn activates the p53 pathway and suppresses the growth of AML cells [58].

### 2.4. Constitutive Photomorphogenic-1 (COP1, also Known as RFWD2)

COP1 is a RING finger containing protein and a Ub E3 ligase that acts as a tumor suppressor [59] and oncoprotein [60,61]. COP1 is associated with myeloid differentiation; it is a Ub E3 ligase of C/EBPα that promotes degradation of C/EBPα, and blocks granulocyte differentiation in 32D cells [62]. COP1 has four alternative splicing forms including the wild-type (full length), Δ4 (lacking four amino acids coded in exon 4), Δ20 (lacking 20 amino acids coded in exon 7), and Δ24 (lacking both exons 4 and 7) [63]. Among the four alternative splicing forms of COP1, only Δ4 is able to downregulate C/EBPα. Blocking of myeloid differentiation by COP1 is highly dependent on Tribbles pseudokinase 1 (TRIB1). The TRIB1 binds to COP1 to enhance its Ub E3 ligase activity for C/EBPα, thereby accelerating blocking of the myeloid differentiation of hematopoietic cells for tumorigenesis [62].

### 2.5. F-Box and WD Repeat Domain Containing 4 (FBXW4)

FBXW4 is a subunit of the SCF complex and one of the FBXW proteins. The transcriptional expression profiles of *FBXW* family proteins were determined in 14 AML cells by cells by applying the European Bioinformatics Institute (EMBL-EBI) database [64]. The results showed upregulation of *FBXW2*, *FBXW4*, *FBXW5*, *FBXW7*, *FBXW8*, *FBXW9*, and *FBXW11* [64]. The amount of *FBXW* mRNA expression in AML patients was analyzed using the Gene Expression Profiling Interactive Analysis (GEPIA) computer tool and The Cancer Genome Atlas (TCGA) database, and *FBXW4* was observed to be upregulated in many AML patients [64]. Older patients showed higher expression of *FBXW4*, and the overall survival (OS) and events-free survival (EFS) were shorter in the high expression group [64]. In a GSEA analysis of the RNA-seq data, AML patients with high *FBXW4* expression showed significantly enriched gene expression of enhancer of zeste homolog 2 (EZH2), histone deacetylase 7 (HDAC7), and lysine demethylase 1A (KDM1A), which are all key epigenetic regulators with cellular functions in tumorigenesis [64]. These data suggest that FBXW4 may mediate degradation of epigenetic proteins in AML through the assembly of Ub E3 ligase SCF.

### 2.6. F-Box and WD Repeat Domain-Containing 7 (FBXW7, AGO, or hCDC4)

FBXW7 is one of the F-box proteins, which acts as a tumor suppressor through ubiquitination and inducing the degradation of numerous important transcription factors and proto-onco proteins in numerous human cancer, including leukemia [65]. Based on the Oncomine database (https://www.oncomine.org, accessed on 5 January 2020), *FBXW7* expression is reported to be higher in leukemia than in other malignancies and normal tissues [65]. Glycogen synthase kinase 3β (GSK3β) is a serine/threonine kinase that is overexpressed in cancers (including AML), where it promotes self-renewal, growth, and survival of malignant cells [66]. Hence, GSK3β inhibition represses AML cell growth and promotes myeloid differentiation. Master transcription factor purine rich box-1 (PU.1) of the monocyte-macrophage differentiation pathway is a potential GSK3β target [67]. Furthermore, the GSK3β phosphorylation consensus motif (S/TXXXS/T) often coincides with the FBWX7 phosphodegron motifs [68]. GSK3β phosphorylates PU.1 at serine41 and serine140, leading to its recognition and subsequent ubiquitin-mediated degradation by Ub E3 ligase FBXW7 [69].

### 2.7. F-Box Protein 9 (FBXO9)

FBXO9 is one of the F-box proteins, which are subunit of the SCF complex. Microarray expression analysis of 898 patients, including 351 normal karyotype AML patients, showed low expression of *FBXO9* among the F-box proteins [70]. Patients with decreased *FBXO9* expression had a poorer prognosis and shorter survival periods than patients with higher expression levels [70]. Knockout of *FBXO9* in the murine hematopoietic system using the CRISPR/Cas9 system showed no effect on stem and early progenitor cells, but resulted in noticeably accelerated and aggressive development of leukemia in the mouse AML model with inversion 16 (p13q22) [70]. *FBXO9* not only plays a role in leukemia initiation, but also functions to maintain AML activity and promote disease progression [70]. The results of quantitative liquid chromatography-tandem mass spectrometry (LC-MS/MS) analysis from primary splenic tumors revealed that tumors lack the FBXO9 proteins associated with cell growth and metastasis (SERPINB1, ADK, ARF1, UAP1L1, CAPG, FMNL1, PAPSS2, USP5, and GOLPH3) [70]. Moreover, the loss of *FBXO9* promotes proteasome activity and sensitizes it to proteasome inhibitors such as bortezomib [70].

### 2.8. RING Finger Protein 5 (RNF5)

RNF5 is a Ub E3 ligase, anchored to the endoplasmic reticulum (ER) membrane and crucial components of the ER-associated degradation (ERAD) process [71]. Based on the Cancer Cell Line Encyclopedia (CCLE) and TCGA database, mRNA expression level of *RNF5* is abundant in AML compared to other cancer types, and there is a positive correlation between high *RNF5* expression and poor survival [72]. Furthermore, protein expression levels of RNF5 in AML cells and peripheral blood mononuclear cells (PBMCs) from independent cohorts of AML patients are higher than other cancer types and control samples [72]. Knockdown of *RNF5* using *shRNF5* in AML cell lines (MOLM-13 and U937) and human AML xenograft model resulted in decreased viability and proliferation [72]. LC-MS/MS analysis revealed that RNF5 interacts with the histone-binding protein retinoblastoma-binding protein 4 (RBBP4) [72]. RNF5 ubiquitinates RBBP4 and regulates the gene expression mediated by RBBP4, but does not regulate stability of RBBP4 [72].

### 2.9. SCF^S-phase kinase–associated protein 2^ (SCF^Skp2^)

Skp2 is an F-box protein, constituting one of the four subunits of the SCF complex [73]. A transcription factor C/EBPα is a crucial factor for hematopoietic stem cell maintenance, inhibition of the leukemia maintenance program, and induction of myeloid differentiation [74]. The Ub E3 ligase SCF^Skp2^ binds to and ubiquitinates C/EBPα which in turn, leads to its degradation through the ubiquitin-proteasome system [75]. Also, SCF^Skp2^ was determined to downregulate the transactivation potential and DNA binding ability of C/EBPα in an AML cell line [75].

CDK2 regulates the cell cycle and influences diverse biological processes including DNA damage, intracellular transport, protein degradation, and signal transduction [66]. Inhibition of CDK2 downregulates SCF^Skp2^and stabilizes the C/EBPα protein expression [76]. CDK2 phosphorylates and stabilizes SCF^Skp2^, which in turn promotes ubiquitination and degradation of C/EBPα [77]. Furthermore, knockdown of *Skp2* abolishes the CDK2-mediated degradation of C/EBPα, resulting in the inhibition of C/EBPα activity and promotion of myeloid differentiation [77].

### 2.10. Two RING Fingers and Double RING Finger Linked (DRIL) 1 (Triad1)

Triad1 is an anti-proliferative Ub E3 ligase and induces apoptosis by enhancing p53 transactivation [78]. Chromosomal translocations associated with the *MLL1* gene characterize a poor prognosis subset of AML [79]. *MLL1* fusion protein (Mll-Ell) results in increased expressions of *HoxA9*, *HoxA10*, and *Triad1*. Mll-Ell activates the *ARIH2* promoter that encodes the *Triad1* in a HoxA9/HoxA10-dependent manner [80]. HoxA9 and HoxA10 have conserved DNA-binding domains and are reported to regulate the growth factor FGF2 [81]. FGF2-induced proliferation is remarkably increased by *Triad1* knockdown but decreased by *Triad1* overexpression. The in vivo knockdown of *Triad1* results in enhancing the Mll-Ell-induced leukemic myeloproliferation and promotes progression to AML [80].

### 2.11. WW Domain-Containing E3 Ubiquitin Protein Ligase 1 (WWP1)

WWP1 is a Ub E3 ligase associated with the progression of numerous epithelial cancers [82]. In bone marrow mononuclear cells (BMMNCs) obtained from AML patients, the *WWP1* gene expression is much higher as compared to healthy donors [83]. WWP1 is an E3 ligase for p27^Kip1^ which mediates the K48-linked polyubiquitin chain and subsequent proteasomal degradation of p27^Kip1^ [83]. Inactivation of WWP1 in AML cells rapidly induces G_0_/G_1_ arrest, which is preceded by post-translational stabilization of p*27*^Kip1^ [83]. Interestingly, *WWP1* gene silencing using *shWWP1* inhibits cell proliferation in AML cell lines and primary cultures of AML, and induces differentiation of AML cells through autophagy signaling [83].

E2 and E3s are associated with pathogenesis of the AML (Table 3), which is involved in overall survival, drug resistance, proliferation, and differentiation. This suggests that E2 and E3 inhibitors could potentially be used for therapeutic strategies in AML.

## 3. Deubiquitination in AML

Deubiquitination is the reverse process of ubiquitination, is mediated by DUBs. DUBs are the most studied DCEs comprising of: ubiquitin-specific proteases (USP), ubiquitin C-terminal hydrolases (UCH), otubain domain ubiquitin-binding proteins (OTU), Machado–Joseph disease protein domain proteases (MJD), the MIU-containing novel DUB family (MINDY), the monocyte chemotactic protein-induced proteins (MCPIP) families, the Zn-finger and UFSP domain protein (ZUFSP) family, the permuted papain fold peptidase of dsDNA viruses and eukaryotes (PPPDE), and the Jab1/MPN domain-associated metalloisopeptidase (JAMM) family [84,85]. Depending on their enzymatic cleavage mechanism, DUBs are classified into two types: cysteine protease and metalloprotease. The cysteine protease DUBs include the remaining eight subfamilies, excluding the JAMM family; they cleave the isopeptide bond of lysine residues ubiquitinated by catalytic cysteine [86]. All metalloprotease DUBs are involved in cleaving the isopeptide bond of lysine residues by a catalytic serine and a zinc ion cofactor [87,88].

### 3.1. Ubiquitin-Specific Protease 3 (USP3)

USP3 is a member of the ubiquitin-specific protease (USP) family. USP3 deubiquitinates the K63-linked polyubiquitin chains on RIG-I and downregulates type I interferon signaling [89]. USP3, also deubiquitinates p53 and stabilizes the p53 protein [90]. Microarray and ChIP-seq data analyses were performed to identify upregulated genes during TPA-induced AML cell differentiation. In AML cells, levels of *USP3* are increased after exposure to TPA, resulting in the reduction of H2AK119ub levels [91]. Thus, USP3 plays an important role in TPA-induced AML cell differentiation by regulating H2AK119ub [91].

### 3.2. Ubiquitin-Specific Protease 7 (USP7 also Known as HAUSP)

USP7 is a member of the USP family, and one of the most studied DUBs. USP7 participates in the regulation of apoptosis through modulation of the p53-dependent and p53-independent pathways [92]. USP7 deubiquitinates p53 or mouse double minute 2 (MDM2), an E3 ligase that ubiquitinates p53 for proteasomal degradation [92]. It has been demonstrated that annexin-1 (ANXA1) and nucleolin bind to HAUSP through their binding motifs. HAUSP deubiquitinates and stabilizes ANXA1 and nucleolin [92,93].

Knockdown of *USP7* using *siUSP7* in the AML cell line HL-60 cells results in decreased cell proliferation and viability. Injecting OCI-AML3 cells into the NOD SCID gamma mouse (NSG mouse) and subsequent treatment with the USP7 inhibitor (P22077) improves mouse viability [94]. Checkpoint kinase 1 (CHK1) is a prognostic marker of AML, and its overexpression is related to resistance to cytarabine. It is reported that USP7 and CHK1 are associated with the highly expressing CHK1 AML cells [94]. USP7 interacts with and stabilizes the CHK1 protein by removing K48-linked polyubiquitin chains from CHK1 in AML cells [94]. Inhibition of USP7 using USP7 inhibitor (P22077) sensitizes AML cells to chemotherapy (cytarabine) [94]. These data indicate that USP7 increases drug resistance through stabilizing CHK1.

USP7 binds to and deubiquitinates the phosphatase and tensin homolog deleted in chromosome 10 (PTEN) and cytoplasmic NPM (NPMc+) [95,96]. In the nucleolus, NPM wild-type binds to USP7, thereby preventing interaction between PTEN and USP7. In the cytoplasm, NPMc+ binds to HAUSP and prevents the deubiquitination of PTEN by USP7. As a result, PTEN remains in the cytoplasm, resulting in its functional loss [95].

### 3.3. Ubiquitin-Specific Protease 9 X-Linked (USP9X)

Located on the X-chromosome, USP9 is a member of the USP family [97]. Recent reports state that USP9X promotes or suppresses tumorigenesis in an environment-dependent manner [98,99]. Treatment of leukemic cell lines with DUB inhibitors (i.e., WP1130, G9, and P22077) revealed that exposure to WP1130 results in noticeably downregulated proliferation and induced apoptosis of MV4-11, a human leukemic cell line harboring FLT3-ITD. USP9X binds to FLT3-ITD and removes its K63-linked polyubiquitin chains [100]. Binding of FLT3-ITD leads to tyrosine phosphorylation and ubiquitination of USP9X and induces its proteasomal degradation [100]. Inhibition of USP9X by applying an inhibitor (WP1130) induces apoptosis through oxidative stress, and activates the stress-related MAP kinase pathways and DNA damage responses.

### 3.4. Ubiquitin-Specific Protease 10 (USP10)

USP10 is a member of the USP family, and is involved in the deubiquitination of p53, Krüppel-like factor 4 (KLF4), NOTCH1 intracellular domain (NICD1), Yes-associated protein (YAP), transcriptional coactivator with PDZ-binding motif (TAZ), AMP-activated protein kinase (AMPK), and Smad4 [101,102,103,104,105,106]. FLT3 regulates hematopoiesis and approximately 30% AML patients are *FLT3*-mutated [107]. USP10 deubiquitinates both FLT3-ITD (the most common FLT3 mutation) and wild-type FLT3 [107]. Dual treatment of FLT3-ITD-expressing Ba/F3 cells, MOLM13-luc+ cells, and MOLM14 cells with HBX19818 and an FLT3 kinase inhibitor resulted in decreased cell proliferation, as compared to treatment with either agent alone [108]. Inhibition of USP10 may offer a strategy for targeting mutant-FLT3 AML and has the capability to overcome kinase-inhibitor resistance [108].

### 3.5. Ubiquitin-Specific Protease15 (USP15)

USP15 is a DUB that belongs to the USP family and is involved in numerous functions. USP15 deubiquitinates the Ub E3 ligase MDM2, eukaryotic initiation factor 4A-I (EIF4A1), terminal uridylyl transferase 1 (TUT1), and FK506-binding protein 5 (FKBP5) [109,110,111,112,113]. TRAF-interacting protein with a forkhead-associated domain B (TIFAB) has been implicated in various cellular signaling pathways associated with hematopoietic and immune cells [114]. TIFAB binds to the catalytic domain of USP15 resulting in improved activities such as deubiquitination of MDM2 (a Ub E3 ligase of p53) [115]. Deletion of TIFAB increases p53 signaling which in turn decreases leukemic cell function and development of leukemia in vivo [115].

Tet methylcytosine dioxygenase 2 (TET2) is a member of the ten–eleven translocation (TET) protein family that regulates cell fate decisions during development and in embryonic stem cells by maintaining pluripotency or by regulating differentiation through catalyzing the demethylation of 5-methylcytosine on DNA [14]. Loss-of-function mutations of *TET2* occur frequently in malignancies of both the myeloid-specific AML and lymphoid lineages, such as angioimmunoblastic T cell lymphoma [116]. TET2 promotes its function by monoubiquitinating at lysine1299, whereas USP15 deubiquitinates lysine1299-linked monoubiquitin and downregulates the TET2 activity [110].

### 3.6. Ubiquitin-Specific Peptidase 22 (USP22)

USP22 is upregulated in different cancer types and is associated with poor prognosis in numerous malignancies [117,118,119,120]. USP22 is part of the Spt-Ada-GCN5-acetyltransferase (SAGA) complex that is linked to cancer progression [121]. *RAS* mutations are commonly observed in juvenile myelomonocytic leukemia (JMML) and chronic myelomonocytic leukemia (CMML), both being subtypes of myeloid leukemia, that transform into acute AML in about 10% and 50% patients, respectively [122]. Moreover, approximately 15–25% AMLs harbor activating mutations in *NRAS* or *KRAS* [123]. Transgenic mouse models with genetically accelerated demise showed abrogated USP22 blocked myeloid differentiation, increased expression of Myc targets genes, and reduced expression of the PU.1 target genes [122]. Expression level of PU.1 was decreased in USP22-deficient KRAS-induced myeloproliferative neoplasm mice (KMUKO) as compared to the KRAS-induced myeloproliferative neoplasm mice (KM) [122]. USP22 binds to and deubiquitinates PU.1, positively regulates PU.1 protein stability and promotes the expression of PU.1 target genes [122]. These data indicate that USP22 plays an important role in myeloid differentiation subsequent to oncogenic KRAS activation.

### 3.7. Ubiquitin-Specific Peptidase 28 (USP28)

USP28 acts as a crucial regulator of cell proliferation through deubiquitinating c-Myc, FBW7, c-JUN, and LSD1 [124,125,126]. According to mass spectrometry analysis using Flag-tagged uridine-cytidine kinase 1 (UCK1) transfected HEK293T cells, the Kelch like family member 2 (KLHL2) and UCK1 interact and bind directly [127]. KLHL2 is a Ub E3 ligase of UCK1 which induces a K48-linked polyubiquitin chain at K81, and promotes degradation [127]. Knockdown *KLHL2* using *shKLHL2* increases 5′-AZA sensitivity for AML cells [127]. As a result of screening to find DUBs of UCK1 from among the 45 known mammalian DUBs, only USP28 activated the luciferase activity of UCK1 [127]. In contrast to KLHL2, USP28 deubiquitinates UCK1 and improves the stability [127]. USP28 does not bind directly with UCK1, but binds via KLHL2 [127]. These data indicate that USP28 decreases the 5′-AZA sensitivity by deubiquitinating and stabilizing the UCK1.

These DUBs are associated with the pathogenesis of the AML (Table 4), which is involved in drug resistance, proliferation, and differentiation. This suggests that DUB inhibitors could be applied to therapeutic strategies in AML.

## 4. Proteins Involved in Regulating the Activity of the Ubiquitination Machinery in AML

### 4.1. Cyclin-Dependent Kinase 2 (CDK2)

CDK2 downregulates wild-type, isoform, and phospho-deficient mutants of *C/EBPα* [76]. CDK2 also inhibits its transactivation potential and cellular functions [76]. It appears that CDK2 phosphorylates a few Ub E3 ligases of C/EBPα, which subsequently degrade it [76]. Among the several CDKs, CDK2 is specifically degraded through ubiquitin-dependent proteasome degradation during AML cell differentiation [128]. In AML cells, *CDK2* knockdown upregulates the expression levels of CD11b and PU.1 and induces myeloid differentiation [128]. *CDK2* knockdown suppresses tumor growth, leads to differentiation in human AML xenograft models, and extends the survival of NOD/SCID mice inoculated with AML cells [128].

### 4.2. TRIB1 and Tribbles Pseudokinase 2 (TRIB2)

Mammalian TRIB1, TRIB2, and Tribbles pseudokinase 3 (TRIB3) contain a highly conserved pseudokinase domain flanked by the N-terminal extension and the C-terminal tail [129]. In spite of their structural significant similarity, TRIB1 and TRIB2 induce AML in mice receiving bone marrow (BM) transplantations [130], downregulate C/EBPα in a proteasomal dependent manner [130], promote self-renewal in hematopoietic progenitors [130], and transduce 32D cells efficiently to block the C/EBPα-dependent 32D cell differentiation; these effects are not exerted by TRIB3 [130]. Moreover, by replacing the C-terminus of *TRIB3* with *TRIB1* or *TRIB2*, the C-terminal swap mutants of *TRIB3* are unable to efficiently degrade C/EBPα and induce AML [130]. This is due to the fact that the functional differences of TRIB1 and TRIB2 are mapped to the kinase domain (KD) rather than the N- or C-terminus [130].

### 4.3. TRIB3

TRIB3 is a mammalian homolog of Drosophila tribbles 3, and is expressed in many tissues, including the liver, adipose, and heart [131,132,133]. Compared to normal human hematopoietic stem cells (HSCs), *TRIB3* mRNA expression is upregulated in AML patients [134]. In AML cells, a knockdown of *TRIB3* using *siTRIB3* increases expression levels of the apoptosis-related proteins, cleaved caspase-3 and cleaved PARP [134]. Similarly, FACS analysis revealed elevated apoptosis rates in the AML cell lines (KG1a and THP-1) treated with *siTRIB3*, as compared to the negative control (siRNA-NC) [134]. Although there are no known specific Ub E3 ligases that promote the ubiquitin-mediated degradation of peroxisome proliferator-activated receptor-α (PPARα), TRIB3 induces ubiquitin-proteasomal degradation of PPARα to reduce the protein stability, thereby inhibiting the apoptotic function of PPARα in AML [134].

## 5. SUMOylation in AML

Non-covalent interactions of SUMO with substrates are mediated by amino acid sequences termed SUMO-interacting motifs (SIMs) that have been identified in numerous proteins including SUMO E3 [135]. SUMO E1 contains two subunits including SUMO E1 (SAE1 or Aos1) and SUMO E2 (SAE2 or Uba2) [33]. SAE1 (Aos1) catalyzes the formation of SUMO-AMP, after which SUMO is transferred to SAE2 (Uba2), and subsequently to UBC9, which is the only one SUMO E2 [33]. Finally, UBC9 and SUMO E3 promote an isopeptide bond between the SUMO and a lysine residue of the target protein [33].

### 5.1. Ubiquitin-like Modifier Activating Enzyme 2 (UBA2, also Known as SAE2)

RNA sequencing of aggressive AML patients and fusion transcripts were analyzed using the SOAPfuse project, wherein the *UBA2-WTIP* fusion gene was identified [136]. To verify the results, RT-PCR was performed in a cohort of 56 clinical AML patients. Of the 56 clinical AML patients assessed, the *UBA2-WTIP* fusion transcript was found in 19 patient samples [136]. The UBA2-WTIP fusion promotes leukemic cell proliferation both in vivo and in vitro through phosphorylation of STAT3, STAT5, and ERK1/2 [136].

### 5.2. Ubiquitin Conjugating Enzyme 9 (UBC9)

Insulin-like growth factor 1 (IGF-1) is secreted in response to growth hormone stimulation, and plays an important role in cell proliferation and development [137]. IGF-1 binds to insulin-like growth factor 1 receptor (IGF-1R), a transmembrane tyrosine kinase receptor [137]. IGF-1R plays an important role in apoptosis, angiogenesis, tumor proliferation, and metastasis [138]. IGF-1R is highly expressed in both AML cell lines and clinical samples of AML [139]. IGF-1 stimulates cell proliferation by inducing IGF-1R expression [138]. Lysine1025 and lysine1100 are identified as sites of SUMOylation on IGF-1R [140]. AML cells transfected with the SUMOylation motif of IGF-1R mutants (K1025A and K1100A) result in decreased cell proliferation by IGF-1 [141]. Ubiquitin conjugating enzyme 9 (UBC9) is a SUMO E2-conjugation enzyme and plays a critical role in SUMOylation [142]. Inhibition of UBC9 using an anti-UBC9 antibody reduces the expressions of IGF-1R and SUMO-1, with subsequent inhibition of cell proliferation [141].

### 5.3. Chromobox Protein 4 (CBX4)

CBX2, one of the polycomb-group (PcG) proteins, is upregulated in several tumors [143] and is associated with lower overall survival [144]. The histone deacetylase (HDAC) inhibitor, suberoylanilide hydroxamic acid (SAHA), downregulates Chromobox protein 2 (CBX2) through SUMOylation at lysine60, lysine153, and lysine410 of CBX2 [145]. Moreover, SUMOylation of CBX2 promotes its polyubiquitination and induces proteasome-mediated degradation [145]. Results of mass spectrometry-based quantitative interaction proteomics show that CBX2 interacts with several proteins including CBX4 (SUMO E3 ligase) [145]. CBX4 induces CBX2 polySUMOylation and its degradation. Since one function of CBX2 is to maintain hematopoietic stem and progenitor self-renewal [146], knockdown of *CBX2* using *shCBX2* in leukemic cells results in low proliferation [145].

### 5.4. Homeodomain-Interacting Protein Kinase 2 (HIPK2)

HIPK2 has diverse functions in transcriptional regulation during development, cell fate determination, induction of apoptosis, and DNA damage responses [147]. The SUMO-interacting motif (SIM) function of HIPK2 is critical for the HIPK2-induced serine46-phosphorylation of p53 and p53-induced apoptosis [148]. *HIPK2* mutations (R861W and N951I) were identified in AML and myelodysplastic syndrome (MDS) patients, with remarkably less phosphorylated p53 at serine46 as compared to wild-type HIPK2 [149]. HIPK2 mutants sequestrate the a nuclear matrix-associated transcription factor essential for hematopoiesis (AML1b) [150], out of the promyelocytic leukemia nuclear bodies (PML-NBs) which are dynamic sub-nuclear structures regulated by post-translational modifications [151], thereby resulting in the dysfunction of AML1b-mediated transcriptional activation and differentiation [149].

### 5.5. Short-Form Positive Regulatory Domain I-Binding Factor 1 and Retinoblastoma-Interacting Zinc Finger Protein-1 (sPRDM16)

The *PRDM16* locus encodes 2 isoforms: the full-length PRDM16 (or MEL1), and the short isoform, sPRDM16 (or MEL1S) [152]. Isoforms depend on the existence of the PR domain (a 134–amino acid region with homology to the SET domain) and the structural hallmark of histone methyltransferases at their N-terminus [152]. Overexpression of SPRDM16 in THP-1 cells promotes proliferation, enhances the self-renewal capacity, and inhibits cellular differentiation. SPRDM16 is SUMOylated by SUMO1 on lysine568, and SUMOylation of sPRDM16 promotes tumorigenesis in AML [153]. The results of gene ontology using overexpressed sPRDM16-K568R mutant AML cells exhibit a distinct gene expression profile from sPRDM16 subsequent to incubation with phorbol 12-myristate 13-acetate (PMA), which induces differentiation of THP-1 cells [153]. SUMOylation of sPRDM16 remarkably affects the expressions of genes related to cell proliferation, cell cycle progression, chemotaxis, differentiation, and wound response [153].

These SUMOylation-related proteins regulate AML pathogenesis (Table 5). This suggests that SUMOylation-related enzyme inhibitors can be applied for therapeutic strategies in AML. To date, the SUMO E3 ligases of HIPK2 and SPRDM16 remain unknown.

## 6. DeSUMOylation in AML

DeSUMOylation is the reverse process of SUMOylation and is mediated by the SUMO-specific protease (SENP) family [25]. The SENP family is able to cleave the isopeptide bond between the C-terminus of SUMO and the ε-amino group of the lysine residue in the target protein, thereby promoting the release and recycle of SUMO [24].

### SUMO-Specific Protease 2 (SENP2)

SENP2 is a deSUMOylating enzyme that deconjugates SUMO from SUMOylated proteins [140]. E26 transformation-specific (ETS)-related gene (ERG) an ETS transcription factor that plays important roles in physiological and pathological processes [154]. A high ERG expression is associated with a poor prognosis in AML [155]. The expression of ERG at both mRNA and protein levels is especially high in AML cells [154]. Based on the GEPIA database and the TCGA leukemia dataset, the mRNA expression level of *ERG* is significantly upregulated in AML patients, as compared to the normal controls [154]. Overexpression of *ERG* in AML cell lines promotes cell proliferation and inhibits differentiation [154]. A protein inhibitor of activated STAT 4 (PIAS4) belongs to the PIAS family, is a major SUMO E3 ligase, and induces SUMOylation of ERG [154]. Contrary to the PIAS family (E3 ligases), the SENP family regulates cellular processes, such as gene expression and DNA damage response through deSUMOylation [156]. Among the three SENP members (SENP1, SENP2, and SENP3), only SENP2 binds to and deSUMOylates ERG [154]. ERG SUMOylation stabilizes the ERG protein by reducing ubiquitination of ERG and inhibiting the proteasome-mediated degradation [154]. A mutation of *ERG* SUMOylation sites exhibits less proliferation and inhibits the differentiation of AML cells [154]. These data indicate that ERG SUMOylation is associated to AML pathogenesis.

## 7. NEDDylation in AML

Similar to ubiquitination, NEDDylation is achieved out by an enzymatic cascade involving an E1, E2s, and E3s [32]. NEDDylation is activated by the E1 enzyme comprising an NAE1 and a UBA3, followed by transfer of NEDD8 to one of two E2s such as a UBE2M or a UBE2F [32]. Finally, an E3 ligase transfers the NEDD8 from an E2 to a specific target protein [32].

### Histone Deacetylases 1 (HDAC1)

HDACs play critical roles in the transcriptional regulation of eukaryotic cells [157]. Histone deacetylases (HDAC1-11) expression in the samples of 5 remission AML patients and 5 refractory AML patients showed a significant amount of HDAC1 expression in the refractory AML patient samples [158]. Moreover, the expression level of HDAC1 in multidrug-resistant AML cell lines (HL-60/ADM and K562/A02) is determined to be higher than in the nondrug-resistant AML cell lines (HL-60 and K562) [158]. Overexpression of *HDAC1* in the AML cell line improves doxorubicin resistance; conversely, knockdown using *siHDAC1* lowers doxorubicin resistance [158]. The HDAC1 protein is regulated by NEDDylation and ubiquitination [158]. HDAC1 knockdown using siHDAC1 inhibits tumor growth in the presence of doxorubicin in vivo. These data indicate that HDAC1 contributes to the multidrug resistance of AML and is regulated by NEDDylation and ubiquitination [158].

## 8. ISGylation in AML

ISG15, an inducible interferon stimulated gene (ISG), is one of the Ubls. The members involved in the enzymatic cascade of ISGylation are also induced by type I interferons [159]. Similar to ubiquitination, ISGylation is carried out by an enzymatic cascade involving an E1, an E2, and an E3 [34]. The activation of the ubiquitin-activating enzyme 1-like (UBE1L; also known as UBC7) forms a thioester bond with free ISG15. The ISG15 is then transferred to the E2 enzyme, ubiquitin-conjugating enzyme H8 (UbcH8; also known as UBE2L6) or Ube2E1 [34]. The HECT domain and the RCC1-like domain-containing protein 5 (HERC5), and the estrogen-responsive finger protein (EFP; also known as TRIM25) are ISG15 E3 ligases that catalyze the conjugation of ISG15 with a lysine residue of target proteins [160,161].

### Ubiquitin/ISG15-Conjugating Enzyme E2 L6 (UBE2L6, also Known as UBCH8)

UBE2L6 is an E2 ubiquitin/ISG15-conjugating enzyme that plays a critical role in targeting c-Myc for proteasomal degradation, and an important role in transducing DNA damage signals by interacting with a Ub E3 ligase RING finger protein 8 (RNF8) [162]. Gene expression analysis in primary neutrophils obtained from 98 AML patient samples and normal samples revealed that the *UBE2L6* gene was less expressed in primary AML cells than normal mature granulocytes [163]. Neutrophil differentiation is inhibited with *UBE2L6* knockdown in APL cells [163]. UBE2L6 regulates protein ISGylation in APL cells and ISGylation induces neutrophil differentiation in leukemic cells [137].

## 9. DeISGylation in AML

DeISGylation is the reverse process of ISGylation. The ubiquitin-specific protease 18 (USP18) is a known deISGylase that removes ISG15 from ISG15-conjugated proteins [164]. To date, the types and functions of deISGylase remain unknown.

### UBP43 (Also Known as USP18)

UBP43 removes ISG15 from conjugated proteins [165]. Retinoic acid (RA) promotes leukemic cell differentiation and is related to triggering the PML/RARα degradation [166]. RA treatment upregulates *UBP43, UBE1L, and ISG15* expressions in RA-sensitive, but not RA-resistant APL cells [167]. UBP43 deISGylates and stabilizes the PML/RARα protein [167]. Knockdown of *UBP43* using *shUBP43* represses PML/RARα protein levels and inhibits APL cell growth by destabilizing the PML domain of PML/RARα [167]. This inhibitory effect promotes apoptosis but does not affect the differentiation of APL cells [167]. Conversely, upregulated *UBP43* expression stabilizes PML/RARα protein and inhibits apoptosis [142].

## 10. Small Molecules in AML

Deregulation of Ub and Ubls-related proteins is associated with diverse cellular processes and diseases including AML. Therefore, numerous AML-related inhibitors have been identified (Table 6). Through specific inhibitors, modulation of modifiers could be a potential therapy in AML.

### 10.1. Cytarabine and Doxorubicin

Cytarabine and doxorubicin are chemotherapeutic agents used in the treatment of AML [174,175]. Cytarabine plus doxorubicin treatment results in DNA damage and a remarkable decrease in the total AMPK protein, a master regulator of cellular energy homeostasis, in both HL-60 and KG-1 cells [168]. AML cell lines have distinct basal levels of ubiquitin-proteasome system activity, and doxorubicin is effectively targeting AMPK degradation [168]. Conversely, a side effect of doxorubicin involving the inhibition of AMPK after exposure to the drug is observed in several non-carcinoma cells [176].

### 10.2. TAK-243

TAK-243 inhibits UBA1, which is a Ub E1 enzyme required for initiation of the ubiquitin cascade [177]. UBA1 participates in ubiquitination as well as the NEDD8 pathway for degradation [178]. In AML cell lines and AML patient samples, TAK-243 induces apoptosis and inhibits clonogenic growth with no effect on normal hematopoietic stem cells [169]. The inhibition of UBA1 using TAK-243 reduces the level of ubiquitinated proteins and increases proteotoxic stress and DNA damage stress marker expressions [169]. A phase I trial is in progress to investigate the side effects and determine the optimal dose of TAK-243 in treating AML patients (ClinicalTrials.gov Identifier: NCT03816319).

A genome-wide Clustered regularly interspaced short palindromic repeats (CRISPR)/CRISPR-associated protein 9 (Cas9) (CRISPR/Cas9) knockout screen in OCI-AML2 cells identifies the *BEN domain containing 3* (*BEND3*) as a regulator of TAK-243 sensitivity [179]. BEND3, a mediator for transcriptional repression, interacts with chromatin-modifying complexes and utilizes diverse strategies to induce repressive histones and to alter DNA methylation [180]. *BEND3* knockout confers resistance to TAK-243 in vitro and in vivo [179]. TAK-243 treated *BEND3* knockout cells exhibit little induction of proteotoxic stress (activating transcription factor 4 (ATF4), C/EBP Homologous Protein (CHOP), and phospho-c-Jun N-terminal kinase (p-JNK)), DNA damage (γH2AX), and apoptosis (cleaved Poly (ADP-ribose) polymerase (PARP)) markers [179]. *BEND3* knockout upregulated the ATP-binding cassette efflux transporter breast cancer resistance protein (BCRP; which mediates the resistance of numerous unrelated anticancer drugs) [181,182,183] and reduced the intracellular levels of TAK-243 [179]. There are no reports on the additional functions of BEND3 in AML yet.

### 10.3. MLN4924

Exposure of AML cells to the NEDD8-activating enzyme (NAE) inhibitor MLN4924 (a NEDDylation inhibitor) shows antitumor activities that cause apoptosis. Moreover, MLN4924 was determined to be effective in most primary AML cells [184,185]. A dose-dependent exposure to MLN4924 increases the expression of the active caspase-3 protein and the number of sub G0/G1 cells [170]. Inhibition of NAE by treating with MLN4924 reduces the NEDDylation of cullin, and increases the expressions of cullin-dependent substrates such as p27, chromatin licensing and DNA replication factor 1 (CDT-1), as well as nuclear factor erythroid 2–related factor 2 (NRF-2) [170]. Similarly, MLN4924 treated xenograft mouse models show a reduction in the NEDDylation of cullin, and increased expression of p-IαBα [130].

It is reported that a high expression level of miR-155 is related with *FLT3-ITD* mutations and poor prognosis in AML [186,187]. Compared to untreated AML cells, delivery of miR-155 using transferrin-conjugated nanoparticles (Tf-NP) into AML cells increases the SH-2 containing inositol 5′ polyphosphatase 1 (SHIP1) protein (a direct miR-155 target) and cleaved caspase-3, and reduces the number of colonies [171]. Treatment with MLN4924 in FLT3-ITD AML cells downregulates miR-155 expression through the inhibition of the NF-κB activity [171]. Furthermore, MLN4924 treated AML cells show decreased binding ability between NF-κB and the miR-155 promoter and NF-κB-dependent transcriptional activity, and inhibit the PI3K/AKT pathway [171].

### 10.4. 2-D08

NOX family members transfer electrons across the biological membrane [188]. NOX family is known to function in not only phagocytosis but also biosignaling and apoptotic regulation, by generating superoxide anion (O_2_^•–^) from molecular oxygen at the expense of NADPH [189]. Therefore, NOX family relates to reactive oxygen species (ROS) and oxidative stress [190]. UBC9 is the sole E2-conjugating enzyme in the SUMOylation cascade [191]. 2′,3′,4′-trihydroxy flavone (2-D08) is a small molecular agent and functions by blocking the transfer of SUMO from UBC9 [192]. In the AML cell lines MOLM13 and ML2, 2-D08 exhibits anti-cancer effects, such as inhibiting cell proliferation and inducing apoptosis [172]. 2-D08 also induces ROS accumulation and activates the mitochondrial-mediated apoptosis in AML cells through deSUMOylation of NOX2 [172].

### 10.5. TAS4464

TAS4464 is a highly effective and specific inhibitor of NAE and inactivates cullin-RING E3 ubiquitin ligases (CRLs) [193]. Treatment of TAS4464 in AML cell lines induces apoptosis and activates the intrinsic and extrinsic apoptotic pathways [173]. Moreover, treatment of HL-60 and THP-1 cell lines with TAS4464 reduces the expression of cellular FADD-like IL-1β-converting enzyme (FLICE)-inhibitory protein (c-FLIP), an anti-apoptotic protein, and increases the expression of phorbol-12-myristate-13-acetate-induced protein 1 (NOXA), a B-cell lymphoma 2 (Bcl-2) family, and pro-apoptotic protein [173].

### 10.6. Retinoic Acid (RA)

UBE1L is an E1 enzyme that catalyzes the first activation step in the conjugation of ISG15 [194]. RA promotes leukemic cell differentiation and is related to triggering the PML/RARα degradation [166]. Moreover, exposure to RA upregulates the UBE1L expression in NB4-S1 APL cells, while increasing both ISG15 expression and conjugation. UBE1L induces ISGylation of the PML domain of PML/RARα and causes its repression [195].

## 11. Conclusions

**Figure 2:**
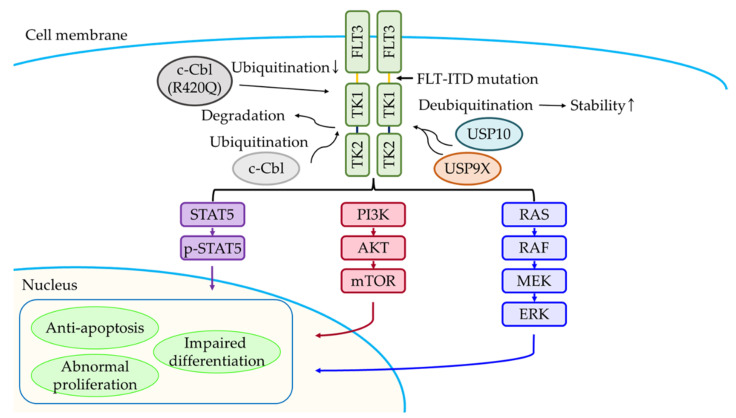
Schematic diagram of the FLT3-ITD signaling pathway. c-Cbl ubiquitinates and induces proteasomal degradation of FLT3-ITD, whereas c-Cbl mutant (R420Q) could not ubiquitinate. USP9X and USP10 deubiquitinate and increase the stability of FLT3-ITD.

**Figure 3:**
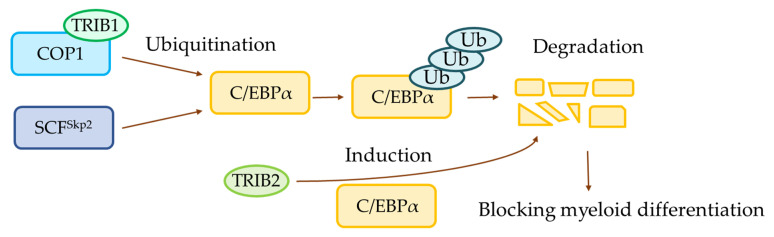
Schematic diagram of the C/EBPα signaling pathway. TRIB1 binds to COP1 and enhances its ubiquitin ligase activity. COP1 and SCF^Skp2^ ubiquitinate and induce proteasomal degradation of FLT3-ITD. TRIB2 binds and promotes proteasome-dependent C/EBPα degradation.

AML is the most common form of acute leukemia and is a malignant disorder of stem cell precursors of the myeloid lineage. AML is highly heterogeneous, with patients exhibiting significant differences with respect to genetic abnormalities, paracrine and autocrine growth regulation, and transcriptional regulation as well as proteomic and cellular metabolomic profiles. Ub and Ubls belong to post-translational modifications, and are involved in cellular functions, such as autophagy, cell-cycle control, DNA repair, signal transduction, and transcription. Substantial researches on AML are currently being conducted, and there are increasing reports indicating that the frequently occurring mutant proteins in AML, such as FLT3 and C/EBPα are related to Ub (Figure 2 and Figure 3). In addition to ubiquitination, SUMO, and NEDD8, there are other lesser investigated Ubls in AML, such as human leukocyte antigen (HLA)-F adjacent transcript 10 (FAT10), autophagy related 12 (ATG12), and ubiquitin-fold modifier 1 (UFM1). We believe that inhibitors or proteins associated with Ub and Ubls can be used as promising molecular targets for the development of therapeutic treatments for AML.

## Figures and Tables

**Table 1:** Common gene mutations in AML.

Genes	Frequency in AML	Functions	References
*FLT3*	FLT-ITD: ~25%	Poor overall survival	[7]
	FLT-TKD: 7~10%		
*NPM1*	~30%	Improved overall survival	[11]
*DNMT3A*	20%	Poor prognosis	[12,13]
*TET2*	10~20%	Variable according to the presence of additional pathogenic events	[15]
*RAS* (*NRAS* and *KRAS)*	10~15%	Aberrant proliferative signaling	[16]
*C/EBP* *α*	~10%	Granulocyte differentiation	[16]

**Table 2:** Protein sequence alignment of Ub and Ubls.

Modifiers	Amino Acid Sequence Identity with Ub (%)	Protein Sizes (kDa)	Amino Acid Numbers	Accession Numbers	References
Ub	100	8.6	76	CAA44911.1	[19]
NEDD8	55	9	81	NP_006147	[32]
SUMO1/SUMO2/SUMO3	18/12/11	11.5/10.8/11.6	101/96/103	NP_001005781/ NP_001005849/ NP_008867	[33]
ISG15	Domain1: 29/ domain2: 31	17	165	NP_005092	[34]
ATG8	14	13.6	117	CAG38511	[26,27]
FAT10	Domain1: 32/ domain2: 40	18.5	165	NP_006389	[28]
UFM1	14	9.9	85	NP_057701	[31]
URM1	13	11.4	101	CAI13492	[31]
SAMP1/SAMP2	21/30	12.9/7.1	87/66	HVO_2619/ HVO_0202	[29]

**Table 3:** Ubiquitination-related enzymes and substrates, and their expressions and functions in AML.

Enzymes	Classification	Target Substrates	Expressions	Functions	References
UbE2E1	E2	-	High	Poor overall survival and increased drug resistance	[51]
c-Cbl	E3	FLT3-ITD	c-Cbl point mutation (Cbl-R420Q)	RTK signaling mitigation	[54]
Cbl-b	E3	SIVA1	-	Inhibition of proliferation	[58]
COP1	E3	C/EBPα	-	Blocking the myeloid differentiation of hematopoietic cells	[62]
FBXW4	E3		High	Mediation of degradation of epigenetic proteins in AML and poor clinical outcome	[64]
FBW7	E3	PU.1	High	Inhibition of monocyte–macrophage differentiation	[68,69]
FBXO9	E3		Low	Poor prognosis	[70]
RNF5	E3	RBBP4	High	Poor survival	[72]
SCF^Skp2^	E3	C/EBPα	-	Myeloid differentiation	[75,77]
Triad1	E3	Mll-Ell	High	Promotion of progression to AML	[80]
WWP1	E3	p27^Kip1^	High	Induction of differentiation	[83]

**Table 4:** Deubiquitination-related enzymes and substrates, and their expressions and functions in AML.

DUBs	Target Substrates	Expressions	Functions	References
USP3	RIG-I	High	Promotion of TPA-mediated leukemia cell differentiation	[91]
USP7	CHK1	-	Increase in drug resistance	[94]
	PTEN and NPMc+	-	Translocation	[95]
USP9X	FLT3-ITD	-	MAP kinase pathways and DNA damage responses	[100]
USP10	FLT3-ITD	-	Kinase-inhibitor resistance	[108]
USP15	MDM2	High	Decrease in p53 signaling	[115]
	TET2		Inhibition of TET2 activity and chemokine expression	[110]
USP22	PU.1	High	Myeloid differentiation upon oncogenic KRAS activation	[122]
USP28	UCK1	-	Decrease in 5′-AZA sensitivity	[127]

**Table 5:** SUMOylation-related enzymes and substrates, and their expressions and functions in AML.

Enzymes	Classification	Target Substrates	Expressions	Functions	References
UBA2	E1	-	UBA2-WTIP fusion	Promotion of leukemic cell proliferation	[136]
UBC9	E2	IGF-1R	-	Caused cell proliferation	[141]
CBX4	E3	CBX2	-	Reduction of proliferation	[145]
-	-	HIPK2	*HIPK2* mutations (R861W and N951I)	Dysfunction of transcriptional activation and differentiation	[149]
-	-	SPRDM16	-	Promotion of tumorigenesis	[153]

**Table 6:** AML-related small molecules, and their target substrates and functions.

Small Molecules	Target Substrates	Functions	References
Cytarabine and doxorubicin	AMPK	DNA damage and decrease of AMPK stability	[168]
TAK-243	UBA1	Reduction of the ubiquitination and increase proteotoxic stress and DNA damage stress marker expressions	[169]
MLN4924	UBA1	Causing apoptosis and inhibition of the NF-κB activity	[170,171]
2-D08	NOX2	Induction of apoptosis	[172]
TAS4464	NEDD8 E1	Induction of apoptosis	[173]
Retinoic acid	NEDD8 E1	Differentiation of leukemic cell	[166]

## Data Availability

Not applicable.
